# Multi-Method Combined Screening of Agarase-Secreting Fungi from Sea Cucumber and Preliminary Analyses on Their Agarases and Agar-Oligosaccharide Products

**DOI:** 10.3390/microorganisms13061235

**Published:** 2025-05-28

**Authors:** Shuting He, Tiantian Lu, Xiaoyu Sun, Fangfang Ban, Longjian Zhou, Yayue Liu, Yan Feng, Yi Zhang

**Affiliations:** 1 Guangdong Provincial Key Laboratory of Aquatic Product Processing and Safety, Guangdong Provincial Engineering Laboratory for Marine Biological Products, Guangdong Provincial Center for Modern Agricultural Scientific Innovation, Shenzhen Institute of Guangdong Ocean University, Zhanjiang Municipal Key Laboratory of Marine Drugs and Nutrition for Brain Health, Research Institute for Marine Drugs and Nutrition, College of Food Science and Technology, Guangdong Ocean University, Zhanjiang 524088, China; 2112203103@stu.gdou.edu.cn (S.H.); lutiantiana@163.com (T.L.); 11211521mml@stu.gdou.edu.cn (X.S.); banfangfang@126.com (F.B.); zhoulongjian@gdou.edu.cn (L.Z.); yayue_liu@163.com (Y.L.); fengfeng1109@126.com (Y.F.); 2 Southern Marine Science and Engineering Guangdong Laboratory (Zhanjiang), Zhanjiang 524088, China; 3 Collaborative Innovation Center of Seafood Deep Processing, Dalian Polytechnic University, Dalian 116034, China

**Keywords:** agarase, fungi, sea cucumber, agar degradation, multi-method precision screening, agar-oligosaccharide

## Abstract

Agar can be degraded into agar-oligosaccharides by physical, chemical, and biological methods, but the further industrial application of agar-oligosaccharides has been limited by the environmental pollution of traditional agar-oligosaccharides preparation methods and the lack of novel agarase. In this study, we reported the screening of 12 strains with agar-degrading activity from sea cucumber intestine and mucus using a combination of Gram’s iodine staining and 3,5-dinitrosalicylic acid (DNS) method, during which five fungal strains exhibited high agarase activity. Their production of different agarases and agar-oligosaccharides could be visualized by zymogram assay and thin-layer chromatography. A strain ACD-11-B with the highest agarase activity showed 99.79% similarity to *Aspergillus sydowii* CBS593.65 for ITS rDNA sequence. Strain ACD-11-B produced five possible agarases with predicted molecular weights of 180, 95, 43, 33, and 20 kDa, approximately. The optimal temperature and pH of the crude enzyme production by strain ACD-11-B were 40 °C and 6.0. The crude enzyme was stable at 30 °C, and Ca^2+^, K^+^, and Na^+^ could increase the activity of the crude enzyme. Its agarases demonstrated remarkable salt tolerance and substrate specificity, with neoagarobiose (NA2) identified as the main degradation product. These results indicate that the fungal strain ACD-11-B can secrete agarases with potential in industrial applications, making it a new producer strain for agarase production.

## 1. Introduction

Agar is one of the major types of marine algal polysaccharides. It is generally extracted from the cell walls of red seaweeds (e.g., *Gelidium* and *Gracilaria*), accounting for about 60% of the dry weight of red algae [1]. Agar is a linear polysaccharide, mainly composed of agarose and agaropectin. Agarose is the main component of agar, accounting for 70% and consisting of 1,3-linked β-D-galactose and 1,4-linked 3,6-anhydro-α-L-galactose. It is mainly responsible for the thickening properties of agar [2]. Agaropectin is an acidic polysaccharide containing sulfate groups, pyruvate, and D-glucuronic acid attached to the branched chain of the agarose β-1,3 bond, which gels poorly. It is the part that commercial extraction seeks to remove [3,4]. Due to agar’s unique gel properties and green and harmless natural characteristics, agar is often widely used as a food additive, thickener, and stabilizer in the pharmaceutical, cosmetic, and food industries. However, the high molecular weight and viscosity of agar and its difficulty in absorption have limited the scope of application of agar. To solve this problem and broaden the application range of agar, most researchers would like to convert agar into agar-oligosaccharides to realize the high-value utilization of agar.

Agarose can be degraded into agar-oligosaccharides (AOSs) and neoagar-oligosaccharides (NAOSs). AOSs are generally the product of acidolysis as well as enzymatic degradation by α-agarase, with D-galactose residues as the non-reducing ends, while NAOSs are usually obtained by β-agarase cleavage, with 3,6-anhydro-α-L-galactose residues as the non-reducing ends [1]. Agar-oligosaccharides have been developed and utilized by numerous researchers due to their small molecular weights, good solubility, and availability for absorption and utilization by the organism [5]. In the past 10 years, agar-oligosaccharides have been successively revealed for their various biological functions, such as prebiotics, antitumor, anti-inflammatory, antioxidant, and neuroprotective activities. They are widely used in the pharmaceutical, food, agriculture, and cosmetics industries. The biological activity of agar-oligosaccharides is closely related to the degree of polymerization (DP) of the sugar, molecular size, type and proportion of monosaccharides, and the anomeric configuration and position of glycosidic bonds [6]. Various agar-derived sugars prepared by multiple enzymatic reactions using an endo-type and an exo-type of β-agarase and a neoagarobiose hydrolase showed prebiotic effects in bifidobacterial fermentation experiments [7]. Oligosaccharides with a higher degree of enzymatic degradation increased antioxidant activity [8]. The neoagarobiose-dominated agaro-oligosaccharides significantly inhibited key pro-inflammatory markers, including nitric oxide (NO), interleukin 6 (IL-6), and tumor necrosis factor-alpha (TNF-α) [9]. The 3,6-anhydro-α-L-galactose, derived from the enzymatic hydrolysis of red algal oligosaccharides, was shown to have anti-colon cancer activity, as it significantly inhibited the proliferation of human colon cancer cells and induced their apoptosis [7]. Agar-oligosaccharides have the potential neuroprotective effects against neurotoxicity induced by 6-hydroxydopamine in SH-SY5Y cells and may reduce neurological damage. The agaro-oligosaccharide monomers, especially agaropentaose, play a major neuroprotective role [10]. Agar-oligosaccharides can not only be used as a novel functional food additive but also have great potential to be developed into clinical drugs for the treatment of certain diseases.

Agar-oligosaccharides can be prepared by physical, chemical, and bio-enzymatic methods. Due to the disadvantages of the physical and chemical method, such as harsh reaction conditions, difficult control of the reaction process, environmental pollution, and destruction of the structure of agar-oligosaccharides, they have been gradually replaced by the bio-enzymatic method, which has become one of the key technologies for the preparation of oligosaccharides [11]. The bio-enzymatic method uses agarases to produce agar-oligosaccharides, with mild reaction conditions and a simple and environmentally friendly process [2]. It ensures high product purity and reduces the negative effect of physicochemical methods on the structural properties of oligosaccharides. Moreover, the agar-oligosaccharides produced by enzymatic degradation have different molecular weights and polymerization degrees and better biological activities. Therefore, the discovery of an efficient and stable agarase plays an important role in realizing the high valorization of agar.

Bacteria able to digest seaweed agar were first isolated from seawater by Glan in 1902. Over the past few decades, agarases have been reported from natural resources, including seawater, marine sediments, seaweeds, marine mollusks, soil, and the human gut [12,13,14,15,16]. The vast majority of producers are bacteria isolated from seaweeds, marine sediments, and seawater, such as *Thalassomonas*, *Alteromonas*, *Microbulbifer*, *Bacillus*, *Zobellia*, *Agarivorans*, *Pseudoalteromonas*, *Catenovulum*, *Halococcus*, *Aquimarina agarilytica*, and *Cellulophaga* [1,17,18]. Besides bacteria, other microbial sources of agarases are still to be explored and exploited. To obtain NAOS or AOS with different DPs from agarose, agarolytic enzymes are needed, including α/β-agarases, α-neoagarobiose hydrolases (NABHs), and agarolytic β-galactosidases [19]. According to the type of glycosidic bond they cleave, agarases are categorized into α-agarases (E.C. 3.2.1.158) and β-agarases (E.C. 3.2.1.81) [20]. Currently, most of the agarases are reported as β-agarases, while there are only nine α-agarases [5]. α-Agarases cleave α-1,3 glycosidic bonds to produce AOS, with the minimal product being an agarobiose (A2). β-agarases cleave β-1,4 glycosidic bonds to release NAOS, with the minimal unit being NA2 [2]. NAOS can be further hydrolyzed by NABHs to generate L-AHG and odd AOS [7]. Another way to classify agarases is based on the glycoside hydrolases (GH) family, which is mainly based on the amino acid sequence similarity of glycoside hydrolases, and more than 130 GH families have been reported so far, including six families of glycoside hydrolases, i.e., GH16, GH50, GH86, GH118, GH117, and GH96 [21]. Agarase has a wide range of applications for the production of high-value-added agar oligosaccharides and for the recovery of DNA from agarose gels. However, commercial agarases have been rare, expensive, and poor performers due to intolerance at extreme pH and heat, which has limited commercial applications. To our knowledge, the only commercial agarase is β-agarases from *Pseudomonas atlantica*, which is very expensive [22]. Therefore, it is necessary to tap a new stable agarase for industrial applications.

Inspired by the phenomenon that sea cucumbers often feed on seaweeds, we hypothesized that some symbiotic microorganisms in their gut could assist their hosts in digesting seaweeds and converting them into nutrients that could be utilized by the organism. To discover novel, valuable producers for agarase preparation and industrial applications, in our previous study, we isolated 18 strains of fungi showing preliminary algal polysaccharide degrading ability from sea cucumber [23]. Herein, we continuously screened their agarase-producing activity and investigated the diversity of agarases and oligosaccharide products, as well as the identification and enzymatic properties of a producer strain with higher activities.

## 2. Materials and Methods

### 2.1. Strains and Materials

The marine fungal strains were isolated from the intestines and mucus of the sea cucumber *Holothuria scabra* collected from the Meizhen sea cucumber aquafarm in Xuwen, Zhanjiang, China. The agar (molecular weight: 3000–9000 Da, cat: A8190), DNS reagent (cat: D7800), and fungal genomic DNA extraction kit (cat: D2300) were from Solarbio Life Science (Beijing, China). Agar oligosaccharides standard (a mixture of NA2–NA10) was purchased from the Qingdao BZ Oligo Biotech Co., Ltd. (Product code: MAA1-3K; Qingdao, China). Sodium dodecyl sulfate-polyacrylamide gel electrophoresis (SDS-PAGE) was prepared using CFAS and a KD PAGE Protein Electrophoresis Gel Preparation Kit Type II (Color, cat: PE008-2) from Shaanxi ZHHC Biotechnology Co., Ltd. (Xi’an, China). All the organic mobile phase solvents for HPLC were from Thermo Fisher Scientific (Waltham, MA, USA). The other reagents were analytically pure.

### 2.2. Preliminary Screening and Identification of Agarase-Secreting Strains

The symbiotic fungi of the sea cucumber have been purified and kept at 4 °C in our previous study. The strains were activated with the Potato Dextrose Agar (PDA) medium and then transferred onto the agar plates of the selective culture medium that contained the following components (g/L): agar, 20; yeast extract, 1; peptone, 2.5; sea salt, 20. The plates were incubated at 28 °C and checked daily until colonies were visible with the naked eye and grew to 1–2 cm; then, the original plates were stained with Gram’s iodine solution. The clear zone around the strain colony indicated that the strain secreted agarase into the medium. The inner and outer diameters of the cleared zone were measured, and the ratio of inner and outer diameters was calculated. The number of strains with clear zones was recorded, and these strains were selected for further analysis [24].

### 2.3. Amplification, Sequencing, and Phylogenetic Analysis of the ITS rDNA

Referring to Lu et al. (2023) [25], the strains showing transparent circles in the experiment described above in Section 2.2 were selected and inoculated onto new PDA plates and cultured at 28 °C. A fungal genomic DNA extraction kit was used to extract the genomic DNA of the strains as templates for PCR amplification of ITS rDNA with an upstream primer (ITS1: 5′-TCGTAGGTGAACCTGCGG-3′) and a reverse primer (ITS4: 5′-TCCTCGCTTATTGATATGC-3′) with the following program: 95 °C for 3 min; (95 °C for 15 s, 56 °C for 15 s, 72 °C for 90 s, repeated 30 times) and 72 °C for 5 min [25]. The amplified PCR fragments were sequenced by Sangon Biotech (Shanghai) Co., Ltd. (Shanghai, China). The sequencing results were aligned with other known strains on NCBI (https://www.ncbi.nlm.nih.gov/guide/all/, (accessed on 20 July 2024)) to determine the strain genus by blastn, and the sequence results were finally uploaded to the GenBank database. Phylogenetic analysis was performed using MEGA version 7.

### 2.4. Agarase Activity Assay

The strains were seeded into 50 mL of semi-solid agar medium consisting of 0.2% agar, 0.25% peptone, 0.1% yeast extract, and 2% sea salt and incubated at 28 °C for 3 days with constant agitation (180 rpm). The fermentation broth was centrifuged at 10,000 rpm for 10 min to acquire the supernatant as crude enzyme solution and then used for the determination of agarase activity. The enzyme activity was measured by the following two different methods.

#### 2.4.1. DNS Method

Agarase activity was determined by reducing sugar production using the DNS method [26]. The crude enzyme solution of 200 µL was added to 800 μL of agar substrate (0.2%, *w*/*v*, dissolved in phosphate-buffered saline (PBS) buffer, pH 7.4), and the reaction was carried out for 30 min at 40 °C. The crude enzyme solution, boiled for 10 min, was used as a control. The reaction was stopped by adding 1 mL of DNS reagent. The mixture was heated at 100 °C for 10 min and then cooled down. The developed color was measured at 540 nm using a microplate reader (Epoch2, Biotek, Winooski, VT, USA). The absorbance values were calculated using D-galactose as a standard. One unit of agarase activity (1 U) was defined as the amount of enzyme that released 1 μg of reducing sugars per minute under the conditions of the proposed assay.

#### 2.4.2. Gram’s Iodine Method

This method takes advantage of the fact that the enzyme can diffuse in the agar, and the agarase activity was defined as the size of the agar area that agarase had degraded. 200 µL of crude enzyme solution was added to an Oxford cup and placed on the surface of the plates with 2% agar. The plates were incubated at 37 °C for 24 h and then stained with Gram’s iodine solution. The diameters of the cleared zone were measured to calculate the total area of the cleared zone. One unit of enzyme activity was defined as the amount of enzyme required to increase the cleared zone area by 0.1 mm^2^/min [24].

### 2.5. SDS-PAGE and Agarase Zymogram Assay

A combination of SDS-PAGE and Zymogram Assay was used to identify and localize the agarases produced by the strains. The crude enzyme solution (about 30 mL) was concentrated by freeze-drying and redissolved to 1 mL with buffer. The precipitate of the crude enzyme solution was removed by centrifugation and then kept at 4 °C. The electrophoretic gel was prepared using a kit, and 25 µL of samples that had been mixed with the loading buffer without β-mercaptoethanol were subjected to 10% SDS–PAGE. The voltage was set to 230 V and stopped when the bromophenol blue indicator reached the bottom of the gel. One gel was stained with Coomassie Brilliant Blue R-250 (CBB R-250) or Fast Silver Stain Kit, and another was stained with Gram’s iodine solution after pretreatment and incubation. The Gram’s iodine staining method of zymogram [27] is as follows: After immersion in 2% Triton X-100 for 30 min to remove SDS and restore the activity of agarase and washing twice with PBS buffer (pH 7.4) to remove Triton X-100, the protein gel was transferred onto a 2% agar plate with 200 µL of PBS and then incubated at 37 °C for 24 h. After incubation, the 2% agar plate was stained using Gram’s iodine solution.

### 2.6. Preparation and Identification of Enzymolytic Products

To determine the enzymolytic products of agarase, the hydrolysis reaction was carried out at 40 °C using 500 μL of the crude agarase solution and 500 μL of the PBS buffer containing 0.2% (*w*/*v*) agar with a reaction time varying from 5 min to 6 h or 48 h. After centrifugation at 7000 rpm for 5 min, the supernatant was collected and mixed with three times the volume of absolute ethanol at 4 °C for 12 h (overnight) and then centrifuged at 5000 rpm for 5 min again to remove the protein and undegraded polysaccharides [28,29]. The supernatant was collected and concentrated by rotary evaporation at 48 °C. The hydrolysates were dialyzed overnight to remove salt and then lyophilized for 24 h. The concentrated hydrolyzed product was redissolved in purified water. The solution was filtered with a filter membrane of 0.22 µm pore size and stored at 4 °C.

#### 2.6.1. Thin-Layer Chromatography (TLC)

The products of agar enzymolysis by crude agarase were analyzed by TLC using a Silica Gel 60 plate (Qingdao Bangkai High-Tech Materials Co., Ltd. (Qingdao, China)). TLC was performed according to the method described by Yan et al. (2020) [30], and the sample loading volume was 5 μL. Enzymolytic products at different times were spotted on a TLC plate, which was developed by 1-butanol/acetic acid/water (2:2:1, *v*:*v*:*v*) as a mobile solvent [30]. To detect oligosaccharides, a visualization solution (0.2% [*w*/*v*] 3,5-Dihydroxytoluene monohydrate, 10% [*v*/*v*] H_2_SO_4_, ethanol) was sprayed on the plates, followed by heating at 120 °C until spots appeared [31]. The mixture of agar oligosaccharides (NA2–NA10) was used as a standard.

#### 2.6.2. High-Performance Liquid Chromatography (HPLC)

Enzymolysis products were analyzed by HPLC-DAD (diode array detector) following derivatization with 1-phenyl-3-methyl-5-pyrazolone (PMP) [32]. An amount of 50 μL of hydrolytic products was added to a 1.5 mL centrifuge tube. Then, 40 μL of 0.5 M PMP (dissolved in methanol) and 50 μL of 0.3 M sodium hydroxide were added to the tube and mixed well. The sealed tube was kept at 70 °C to react for 20 min and then cooled for 10 min. The solution was neutralized with 50 μL of 0.3 M hydrochloric acid, 200 μL of water, and 500 μL of chloroform. After that, the solution was centrifuged at 3000 rpm for 5 min, and the aqueous phase was collected. This procedure was repeated twice to remove redundant PMP.

We carried out the agar oligosaccharide analysis by using HPLC-DAD (Agilent Infinity II 1260, Santa Clara, CA, USA) with a C18 chromatographic column (Kinetex, 4.6 mm × 100 mm, 5 µm). The samples were filtered with a filter membrane of 0.22 µm pore size. The sample injection volume was 20.0 µL. Pure water was used as mobile phase A, and mobile phase B was methanol. The gradient elution condition was as follows: 10% B (0–2.0 min), 10–100% B (2.0–20.0 min), 100% B (20.0–22.0 min), 100–10% B (22.0–22.2 min), and 10% B (22.2–24.0 min). The flow rate was 0.6 mL/min. The HPLC monitoring wavelength was 254 nm. The PMP-derivatized product of the mixture of agar oligosaccharides was used as a reference marker for the HPLC experiments.

### 2.7. Characterization of Crude Agarase Enzyme of Strain ACD-11-B

The strain ACD-11-B was cultured in agar medium by shaking at 28 °C for 72 h and then centrifuged at 10,000 rpm for 10 min to acquire the supernatant as crude enzyme solution, whose activity was tested by detecting the release of oligosaccharides according to the DNS method.

The effect of temperature was studied by measuring the enzymatic activity at several temperatures ranging from 30 to 80 °C in PBS buffer at pH 7.4. Meanwhile, crude enzyme without substrate was incubated for a period of time (0, 1, 2, 3, 4, 5, 6, 12, 24, 36, and 48 h) at several temperatures (30, 40, and 50 °C) to investigate the effect of thermostability by measuring the remaining enzyme activity.

The effect of pH was investigated by measuring the crude enzyme activity at 40 °C in several pH values ranging from pH 3.0 to 11.0 in the buffers with the concentration of 50 mM: Na_2_HPO_4_/citric acid solution (pH 3.0–8.0), Tris-HCl buffer (pH 7.0–9.0), and Gly/NaOH buffer (pH 9.0–11.0). Meanwhile, the effect of pH on the stability was evaluated by measuring the remaining activity of the crude enzyme preincubated at 4 °C for 12 h in several solutions from pH 3.0 to 11.0.

The effects of chemical agents on the activity of the crude enzyme were studied by detecting the activity in the standard assay condition with 10 mM of ethylene diamine tetraacetic acid (EDTA), SDS, and CTAB. Additionally, the effects of different metal ions (Fe^2+^, Zn^2+^, Ca^2+^, Na^+^, K^+^, Mg^2+^, Cu^2+^, and Ba^2+^) at a concentration of 10 mM on the activity of the crude enzyme were also investigated.

The effect of NaCl concentrations on the crude enzyme activity was measured by determining the activity at various NaCl concentrations ranging from 0 to 3 M.

The substrate specificity of the crude enzyme of strain ACD-11-B was determined with agar, alginate, carrageenan, and cellulose as substrates, respectively, at 40 °C in 50 mM Na_2_HPO_4_/citric acid solution (pH 6.0).

### 2.8. Statistical Analyses

In this study, data analysis and processing were carried out using GraphPad Prism 9.0 software. Quantitative data were all presented in the form of mean ± standard deviations, and the significant differences between the groups were determined by one-way analysis of variance (ANOVA) using GraphPad Prism 9.0 software. To ensure the reliability of the experimental results, three independent replicate experiments were set up for all measurements. The visualization processing of the liquid chromatography experimental data was achieved using Origin 2022 software.

## 3. Results

### 3.1. Screening and Identification of Agarase-Secreting Strains

**Figure 1:**
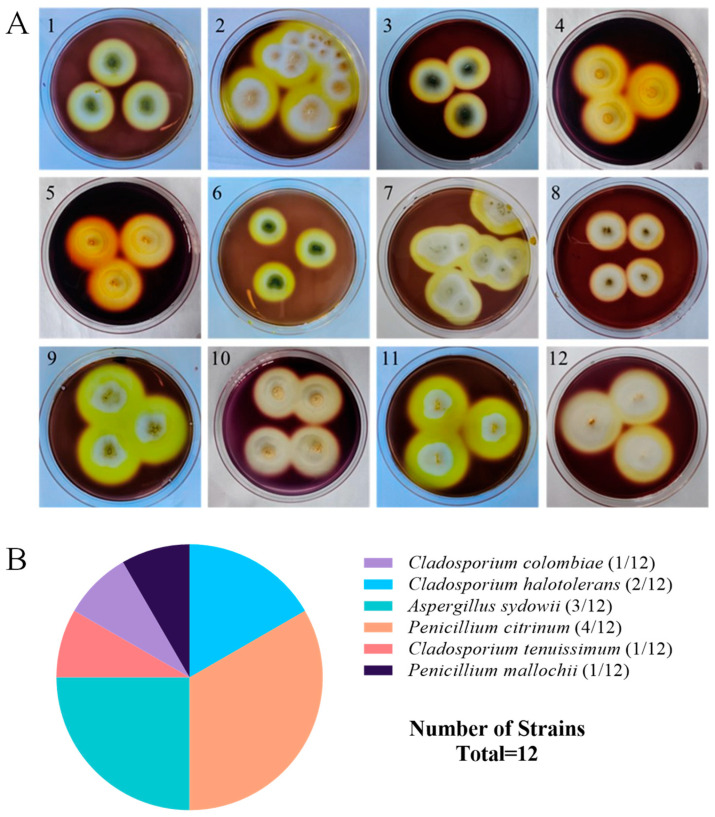
Screening and identification of agarase-secreting strains. (**A**) The strains showed the distinct zones of agar degradation screened by Gram’s iodine staining. Pictures 1–12 are strains NY5-1, NY-13, NY-7, CD-1, ACD-2, ACD-3, ACD-5, ACD-7, ACD-8, ACD-11-Q, ACD-11-B, and ACD-12, respectively. (**B**) Taxonomical distribution of the agarase-secreting fungal strains.

**Figure 2:**
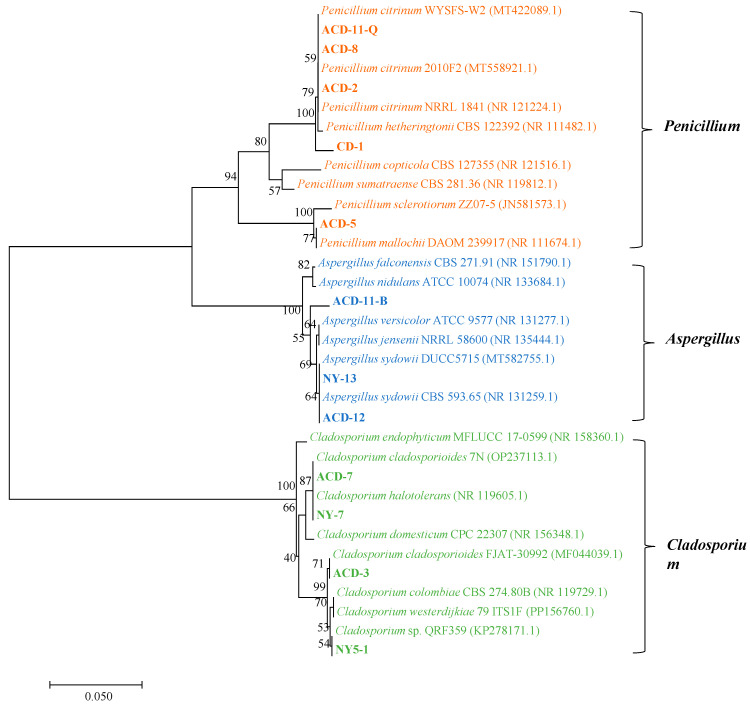
Neighbor-joining phylogenetic tree of the 12 agarase-secreting fungi based on the ITS rDNA sequences. The neighbor-joining method was used to construct the phylogenetic tree using Mega 7 software. (Bootstrap values were calculated using 1000 replications).

A total of 18 strains of symbiotic fungi were isolated from sea cucumber intestinal and mucus samples in the previous study, among which 12 strains showed distinct zones of clearance around their colonies after staining with Gram’s iodine solution (Figure 1A). According to the principle of Gram’s iodine staining, iodine can complex with agar to form a dark purple complexation. The agar area degraded by agarase showed distinct zones of clearance due to the absence of agar-iodine complexation, which indicated that these fungi had agar degradation activity [33,34]. These strains were subjected to ITS rDNA gene amplification. Sequence analysis revealed that these strains with degrading enzyme activity accounted for 66% of the total isolated strains, including five from *Penicillium* sp. (four: *Penicillium citrinum*, one: *Penicillium mallochii*), four from *Cladosporium* sp. (one: *Cladosporium colombiae*, two: *Cladosporium halotolerans*, one: *Cladosporium tenuissimum*), and three from *Aspergillus* sp. (all: *Aspergillus sydowii*) (Figure 1B). Neighbor-joining phylogenetic analysis based on the ITS rDNA gene sequences further confirmed the identity of these 12 strains (Figure 2). The ratio of the diameter of the zones of clearance to the diameter of the colonies (clearance zone/colony ratio) showed that five strains (CD-1, ACD-2, ACD-11-Q, ACD-11-B, and ACD-12) had higher enzyme activity. Their clearance zone/colony ratios reached more than 1.8 (Table 1).

### 3.2. Evaluation of Agarase Activities

**Figure 3:**
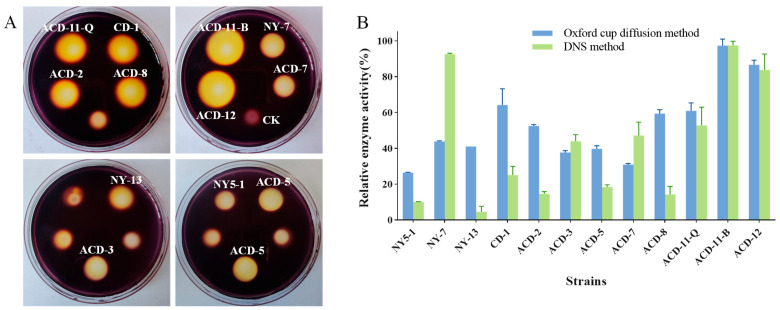
Quantification of agarase activity of the strains fermented in shaking flasks. (**A**) The clearance zones of twelve strains were determined by Oxford cup diffusion method using Gram’s iodine staining (Oxford cups were removed after staining; unlabeled clearance zones were produced by other sea cucumber symbiotic fungi, which had low agarase activities); (**B**) quantification of agarase activities evaluated by Oxford cup diffusion method and DNS method. The enzyme activities of the strain ACD-11-B detected by both methods were taken as 100%, respectively.

Due to the large land area required for solid plate fermentation and the difficulty of agarase extraction by this process, liquid fermentation is still mainly applied for large-scale fermentation of agarase in industry. Usually, different environmental conditions can change the growth patterns and metabolites of microorganisms. The above-mentioned strains were fermented in a semi-liquid medium containing 0.2% agar on a shaker for 72 h, and the crude enzyme solution was extracted by centrifugation. Considering that differences in agarase activity assay methods may influence results, two methods, including the DNS method and the Oxford cup agar diffusion method using Gram’s iodine staining, were combined to accurately re-screen out the strains that can produce agarase under shaking fermentation conditions. As is shown (Figure 3), strains ACD-11-B and ACD-12 showed higher agarase activity, and the results of agarase activity determined by these two methods were consistent with the preliminary screening results of static solid fermentation, while the agarase activity of the other strains determined by different methods deviated. Strains ACD-2, CD-1, and ACD-8 showed higher activities in both the static solid fermentation method and the Oxford cup agar diffusion method, and were less active in the DNS method. In addition, the strains NY-7 and ACD-7 showed opposite results. Furthermore, a few strains like ACD-2 showed significant differences in producing agarase under static solid fermentation (Figure 1) vs. shaking fermentation (Figure 3), as evaluated using Gram’s iodine staining method. It is hypothesized that these phenomena may be related to the method of cultivation of the fungi and the diffusion rate of different-sized agarases they produce in the agar. Combining the above performance, five strains, including ACD-11-B, ACD-11-Q, ACD-12, NY-7, and ACD-7, were selected for further studies.

### 3.3. Profiles of Agarase Produced by the Strains

**Figure 4:**
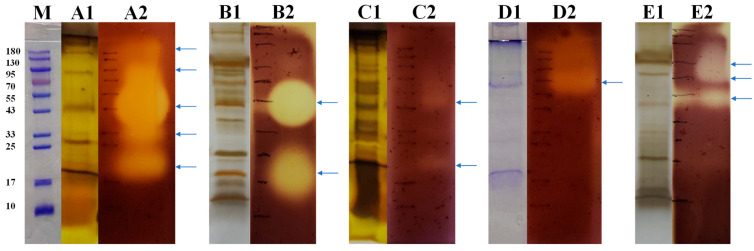
SDS–PAGE and agarase zymogram analysis of extracellular proteins of the strains ((**A1**,**A2**) ACD-11-B; (**B1**,**B2**) ACD-11-Q; (**C1**,**C2**) ACD-12; (**D1**,**D2**) NY-7; (**E1**,**E2**) ACD-7). Lane (**M**), protein marker; for each strain, lane 1: SDS–PAGE of extracellular protein of strain with staining by CBB R-250 (for (**D1**)) or Fast Silver Stain Kit (for (**A1**,**B1**,**C1**,**E1**)); lane 2: zymogram analysis of extracellular protein of strain. The places indicated by the blue arrows represent locations where the strain may produce agarase. After the enzymatic reaction, the agar plates were stained using Gram’s iodine staining.

To reveal the agarase diversity of these strains, we combined SDS-PAGE and zymogram presentation by Gram’s iodine staining to validate the agarases produced by these fungi and display their molecular weight (MW) distribution. As a result, strain ACD-11-B produced the highest richness of agarase with five bands with potent agarase activity (Figure 4), which is consistent with the results of the previous screening. By comparing SDS-PAGE and zymogram, the MWs of the agarases from strain ACD-11-B were determined to be around 180, 95, 43, 33, and 20 kDa, respectively (Figure 4(A1,A2)). Unexpectedly, the strain ACD-11-Q produced two potent agarase bands with MWs of 40 and 20 kDa (Figure 4(B1,B2)), while ACD-12 only produced very weak agarase bands with MWs of 50 and 20 kDa, respectively (Figure 4(C1,C2)). This was contrary to their previous activity in the dual assay by Oxford cup and DNS methods when the crude enzyme solutions were tested as a whole. Strain NY-7 produced one predominant agarase band with an MW of 70 kDa and some faint larger bands (Figure 4(D1,D2)). And strain ACD-7 produced three agarase bands with MWs of 73, 53, and 40 kDa, respectively (Figure 4(E1,E2)). The MW distribution of their agarases is consistent with most of the agarases that have been reported (Table 2).

### 3.4. Agar Enzymolytic Product Analysis of Agarase-Secreting Strains

**Figure 5:**
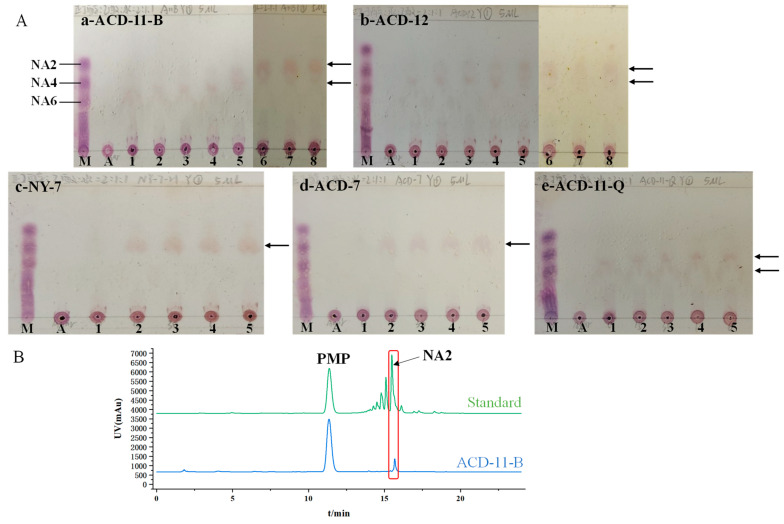
Agar hydrolytic product analysis of agarase-secreting strains. (**A**) TLC analysis of the hydrolysates by the crude agarases of strains ACD-11-B, ACD-12, NY-7, ACD-7, and ACD-11-Q. Lane M: standard marker; lane A: agar without enzyme treatment; lane 1–8: agar with enzyme incubation for 0 min, 15 min, 30 min, 1 h, 6 h, 12 h, 24 h, 48 h, respectively. The places indicated by black arrows represent the locations of the main enzymatic products. (**B**) HPLC analysis of the 24 h hydrolysates by crude agarase of ACD-11-B.

TLC was used to investigate the DP of agar oligosaccharides produced by the agarases of different strains. The five strains (ACD-11-B, ACD-12, NY-7, ACD-7, and ACD-11-Q) with high enzymatic activity under shaking fermentation conditions were selected for the tests. The TLC results show that the crude agarase of strain ACD-11-B hydrolyzed agar, generating various agaro-oligosaccharides during the initial stages of the reaction. With incubation time increasing, the yields of the larger oligosaccharides gradually decreased while that of NA2 increased. After 12 h of incubation, NA2 became the main product (Figure 5(Aa)). The agarases of strain ACD-12 mainly degraded agar as neoagarotetraose (NA4) and neoagarohexaose (NA6). With the prolongation of the digestion time, the end product was NA4 (Figure 5(Ab)). The agarase of strains NY-7 and ACD-7 mainly degraded agar as NA4 (Figure 5(Ac,Ad)). The activity of agarase produced by strain ACD-11-Q was relatively low, and the main products were NA4 and NA6 (Figure 5(Ae)). In summary, the agar degradation products vary depending on the agarases produced by the strains, which provides flexibility for meeting different industrial applications.

To further confirm the agar hydrolysates by the crude enzyme of strain ACD-11-B, the products of 24 h-degradation were identified by HPLC-DAD after PMP derivatization (Figure 5B). The results revealed that the major hydrolytic product by the crude enzyme of strain ACD-11-B had similar retention times to NA2, indicating that NA2 was the main product of the crude enzyme solution of strain ACD-11-B.

### 3.5. Characterization of Crude Agarase Enzyme of Strain ACD-11-B

Since the agarase of strain ACD-11-B showed the highest activity and produced diverse hydrolytic products, the influences of temperature, pH, metal ions, chemical reagents, and salinity on enzymatic activity and substrate specification were investigated to characterize the properties of this strain’s crude agarase.

**Figure 6:**
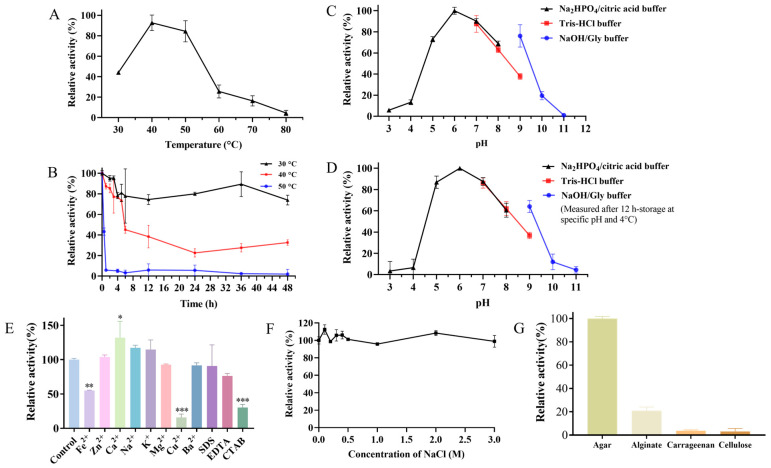
Characterization of the crude agarase of strain ACD-11-B. (**A**) The effect of temperature on the enzymatic activity of crude agarase. The activity was tested from 30 °C to 80 °C. (**B**) The effect of temperature on the enzymatic stability of crude agarase. The residual activity was detected after incubating at 30 °C, 40 °C, or 50 °C at different times (0–6, 12, 24, 36, and 48 h). (**C**) The effect of pH on the enzymatic activity of crude agarase. The activity was tested by incubating crude agarase at 40 °C in these buffers: citric acid/Na_2_HPO_4_ buffer (pH 3.0–8.0); Tris-HCl buffer (pH 7.0–9.0); NaOH/Gly buffer (pH 9.0–11.0). (**D**) The storage stability of crude agarase under different pH conditions. The remaining activity was detected after incubating at 4 °C for 12 h in specific buffers (pH 3.0–11.0). (**E**) The effect of metal ions and chemical agents on the activities of crude agarase. (**F**) Effects of NaCl on the activity of crude agarase. (**G**) Substrate specificity of crude agarase towards agar, sodium alginate, carrageenan, and cellulose. For all of the above plots, the maximal activity of crude agarase was taken as 100%, and other values were indicated as the ratio of the maximum. Data are expressed as SD ± mean (*n* = 3), “*” indicates significant differences between the sample-treated group and the control group (“*” represents *p* < 0.05, “**” represents *p* < 0.01, “***” represents *p* < 0.001).

The effects of temperature on the activity of the crude enzyme of strain ACD-11-B were measured from 30 to 80 °C. The optimum temperature of the crude enzyme was 40 °C (Figure 6A). This is consistent with most of the reported agarases (Table 2). After incubation at 30, 40, and 50 °C for 48 h, the crude enzyme retained 74%, 33%, and 2% of its maximum activity, respectively. The crude enzyme of strain ACD-11-B had a relatively good thermostability at 30 °C. Secondly, the crude enzyme of strain ACD-11-B also remained stable at 40 °C, and the residual activity of the crude enzyme was more than 70% of its maximum activity after incubation at 40 °C for 5 h. However, it basically became inactivated after incubation at 50 °C for 1 h and just retained 45% of its maximum activity after incubation at 40 °C for 6 h (Figure 6B). It is hypothesized that the fungus was isolated from the intestinal tract of a marine invertebrate, and thus, the enzyme is cold-adapted and will show instability at higher temperatures.

The results of the pH effect showed that the activity of the crude enzyme of strain ACD-11-B had an optimum pH of 6.0, with more than 60% of its maximum activity retained in the range from pH 5.0 to 9.0 (Figure 6C). An environment that is too acidic or too alkaline can affect the enzymatic activity. The optimal pH of most of the reported agarases was between 6.5 and 7.5; only a few were higher than 8.0 (Table 2). The results from the pH long-term endurability experiment were similar. The enzyme retained more than 80% of its residual enzyme activity after incubation at pH 5.0–7.0 for 12 h, which reflects that the enzyme is suitable for storage in a neutral or weakly acidic environment. In addition, the enzyme also retained 60% of its residual enzyme activity after incubation at pH 8.0–9.0 for 12 h, which proves that the enzyme is a weakly alkaline-adapted enzyme (Figure 6D). The high activity and stability over a broad pH range make the enzyme more universal for various conditions without pH adjustment, which is suitable for industrial applications.

Enzyme activity is not only affected by pH and temperature but also by metal ions and some chemical agents. Some metal ions and chemical agents are often applied in industrial production, and their residues can affect the enzymatic activity. The effects of chemical agents and different metal ions on the activity of ACD-11-B‘s crude agarase were measured and are listed in Figure 6E. Among these metal ions, Na^+^ and K^+^ enhanced the enzymatic activity of crude agarase by 15%, and Ca^2+^ remarkably promoted the enzymatic activity of crude agarase by more than 30%. However, other metal ions (Fe^2+^, Mg^2+^, Cu^2+^, and Ba^2+^) suppressed the enzymatic activity of crude agarase. Further comparison showed that Mg^2+^ and Ba^2+^ displayed a weak suppression by 7% and 8%, and Fe^2+^ exhibited a moderate suppression by 45%, whereas Cu^2+^ manifested a strong suppression by more than 80%. In addition, those chemical agents (SDS, EDTA, and CTAB) could also reduce the enzymatic activity. Among them, CTAB could also significantly reduce the activity of the crude agarase by more than 75%, but the crude enzyme showed relatively strong tolerance to SDS and EDTA. The above results indicate that strain ACD-11-B’s crude agarase activity is significantly regulated by some metal ions, and the crude enzyme has different tolerances to various common chemical reagents.

For most marine lyases, NaCl of a certain concentration is essential for enzyme activation; the activity was generally significantly lower in the absence of NaCl [35]. As shown in Figure 6F, NaCl slightly activated the crude agarase of ACD-11-B at a concentration of 0–0.5 M and had no effect on the activity at a concentration of 0.5–3.0 M. Thus, the enzyme was basically NaCl-independent and showed stable activity. Due to the NaCl-independent and salt-tolerant properties, the enzyme was competent for a variety of specific processing requirements.

The substrate specificity of the crude enzyme of strain ACD-11-B was further explored by performing enzymatic reactions with agar, alginate, cellulose, or carrageenan as the sole substrate. The enzyme selectively recognized agar as a substrate and carried out enzymatic hydrolysis, showing high substrate specificity. In addition, the enzyme can slightly hydrolyze alginate, while it has no significant hydrolytic effect on cellulose and carrageenan (Figure 6G).

**Table 2:** The biochemical properties of the reported agarases.

Source	Protein	Molecular Weight (kDa)	Optimal Temp./pH	Temp./pH Stability ^a^	Metal Ions/ Chemical Agents ^b^	Main Product	Refs.
*Stenotrophomonas* sp. NTa	N.D. ^c^	89.0	40 °C/10.0	20 °C/30 °C, 1 h, stable; 40 °C, 1 h, 64%; 50 °C, 1 h, 46%; 60 °C, 30 min, no activity; pH 5.0–11.0, >65%;	K^+^, Mg^2+^, Ba^2+^, Ca^2+^, Na^+^	NA2, NA4, NA6	[36]
*Thalassomonas* sp. LD5	AgaD	180	35 °C/7.4	<30 °C/>45 °C, >50%;	Ca^2+^	NA4	[37]
*Vibrio* *Natriegens* WPAGA4	rAga3420	100	40 °C/7.0	10 °C/0 °C, 59.7%/41.2%; 10–30 °C, 50 min, good stability;	Mg^2+^, Fe^2+^, Fe^3+^, Ca^2+^, Mn^2+^, Cu^2+^, K^+^, DTT	NA2	[38]
*Catenovulum* sp. X3	AgaXa	52	52 °C/7.4	<42 °C, >95%; pH 5.0–9.0, 12 h, 85%;	DDT, β-met, Mg^2+^	DP4, DP6, DP8	[39]
*Shewanella* sp. WPAGA9	AgaW1540	50	35 °C/7.0	20 °C, 1 h, 94.7%; 25 °C, 1 h, 97.9%;	Ba^2+^, Mn^2+^, Ni^2+^, K^+^, Ga^2+^, Sr^2+^, Ag^+^, Cr^2+^, Fe^2+^, EDTA	NA4, NA6	[40]
*Alteromonas* sp. SY37-12	N.D.	39.5	35 °C/7.0	50 °C, 15 min, no activity; 70 °C, 1 min, no activity;	Na^+^	NA4, NA6	[41]
*Thalassomonas* sp. LD5	AgaE	97	35 °C/7.0	50 °C, 10 min, no activity;	Na^+^	NA4, NA6	[42]
*D. arenaria* *Nicot*	N.D.	N.D.	40 °C/7.0	40 °C/50 °C; 30 min; >75%; 40 °C/50 °C,1 h, 65.74%/55.46%; pH 4–10, >75%;	N.D.	N.D.	[26]
*Agarivorans* *albus* YKW-34	AgaA34	50	40 °C/8.0	pH 6.0–11.0, 1 h, >80%;50 °C, 1 h, >95%;	β-Me, DTT	NA2, NA4	[43]
*Microbulbifer* sp. BN3	N3-1	34.3	50 °C/6.0	pH 4.0–9.0, >70%; 30–60 °C, 30 min, >40%;	K^+^, Na^+^, Mg^2+^	NA2, NA4	[44]
*Aspergillus* *Sydowii* ACD-11-B	Crude enzyme	N.D.	40 °C/6.0	30 °C, 48 h, >70%; pH 5.9–9.0, 12 h, >60%	K^+^, Ca^2+^, Na^+^	NA2	This study

^a^ The temperature and pH stability: the residual agarase activity after incubation for a certain time at different temperatures and pH ranges. ^b^ Those metal ions increased the enzyme activity at a certain concentration. ^c^ Not determined.

## 4. Discussion

In this paper, we first screened agar-degrading fungi with high and stable activity from sea cucumber by combining multiple enzyme activity assays, including in situ colony iodine staining, the Oxford cup-agar diffusion method, and the DNS method. Five strains with high agarase activity, ACD-11-B, ACD-12, NY-7, ACD-7, and ACD-11-Q, were discovered by this method. Compared with the traditional single screening method, the combination of multiple screening methods has a higher possibility of finding useful enzyme-producing strains for future industrial liquid fermentation, since in some cases, active strains that produce enzymes on solid cultural media do not retain this ability in submerged fermentation [45]. In addition, it made full use of the convenience of Gram’s iodine staining and increased reliability by double-checking with the DNS method, thereby avoiding inaccurate results during the screening process [24].

Then, based on the similarity of ITS rDNA sequences, the 12 strains with agarase activity were identified. Among them, the largest proportion of strains belonged to *Penicillium citrinum*. According to the relevant literature, strains of *Penicillium citrinum* can produce cellulase [46] and lipase [47], while agarase activity is less reported. The strain with the highest agarase activity was identified as *Aspergillus sydowii*. It was reported that this strain has erythromycin-degrading activity, and genomic and transcriptomic analyses also confirmed that strain W1 degraded erythromycin through glycoside hydrolase and esterase activities [48]. Most of the researchers found that the species *Aspergillus sydowii* can produce xylanase efficiently, and Ghosh et al. found that *Aspergillus sydowii* MG49 can produce heat-resistant xylanase (EC 3.2.1.8) and β-xylosidase (EC 3.2.1.37) efficiently using xylan as substrate [49]. Tulsani et al. mentioned that *Aspergillus sydowii* has cellulase, pectinase, and amylase activities in addition to xylanase activity [50]. In addition, it has been reported that this species also possesses keratinase activity [51]; aminopeptidase, a trypsin-like serine protease, and elastin-hydrolyzing serine protease activities [52]; as well as α-galactosidase activity [53]. To our knowledge, the agarase activity of this species is reported for the first time. Moreover, agarases are mainly from marine bacteria and yeast species [54], while fungi are rarely reported as their source. Therefore, this strain has good potential for the development of agarase production.

In this study, the diversity of the extracellular agarases produced by the fungi, as well as their molecular weights, were directly visualized by zymogram assay, which consists of SDS-PAGE and the Gram’s iodine method. This method is intuitive and convenient and can be used as one of the most important methods to track and characterize agarases. However, it has been claimed in the literature that the diverse agarase bands detected by zymogram are not excluded from being shattered by physical shear or protease hydrolysis during experimental processes like long-term incubation [42]. Also, it may cause some inactive zymogens to be activated due to cutting. This possibility also explains that strain ACD-12 only displayed weak agarase bands in the zymogram assay while exhibiting quite strong activity in previous assays using the Oxford cup agar diffusion method and DNS method. It is also a good reason for strain ACD-8′s opposite performance change in the two steps. Therefore, the possible factors activating or inactivating agarases deserve in-depth investigation in future studies. In addition, in this experiment, to balance the accuracy of molecular weight measurement and the recovery of enzymatic activity after electrophoresis, we did not treat the agarase samples with boiling and mercaptoethanol during SDS-PAGE. In the future, native PAGE can be performed to localize the active enzyme bands, and their more accurate molecular weights can be reconfirmed by normal SDS-PAGE of the proteins in bands.

From the results of TLC, the five selected fungi had a simple composition of enzymolytic products from agar, like NA2, NA4, and NA6. According to the related literature, these oligosaccharides have several biological activities and better application prospects. For example, Yu et al. found that agarobiose can be used as a new anticariogenic agent, owing to its anticariogenic activity against *Streptococcus mutans* [55]. Lee et al. discovered a novel β-agarase from the marine bacterium *Gilvimarinus agarilyticus*, which can produce NA4 and NA2 as its main products, and its partial hydrolysis products (PHPs) showed strong hyaluronidase inhibitory activity at a concentration of 1 mg/mL, which has a certain anti-aging effect [17]. NA4 can be used to inhibit the production of the pro-inflammatory cytokine TNF-α and the expression of iNOS [56]. In addition, NA4 can improve cognitive deficits, attenuate amyloid β (Aβ) and Tau pathology, and modulate intestinal flora composition and SCFAs receptor-related pathways in AD mice [57]. A mixture of NA4 and NA6 can inhibit α-glucosidase synthesis [58]. Thus, the selected strains in this study are especially meaningful in producing bioactive agar oligosaccharides. Particularly, the simple composition of products by these fungi can reduce the difficulty of isolation and purification of oligosaccharides.

By stepwise screening, strain ACD-11-B was selected as the producer with the highest agarase activity among the fungal isolates from the sea cucumber. Its crude enzyme exhibited maximum enzyme activity and favorable stability at a relatively low temperature (40 °C), which reduces the energy consumption for the enzymatic reactions. On the other hand, the gel-forming temperature of agar is also around 40 °C. This means an agarase with an optimal temperature of 40 °C can fully contact the agar and maximize its activity [38,39,40].

Secondly, the enzyme has high activity in a neutral to acidic environment, which saves the reagents required for acid-base adjustment. This wide pH range enables the enzyme to flexibly adapt to different pH reaction environments, as well as to pH changes during the reaction process.

Meanwhile, it was found that Ca^2+^ could significantly increase enzyme activity. Strong calcium dependence is common in known agarases. This dependence may be attributed to carbohydrate-binding modules (CBMs) since all calcium-binding sites are located in CBMs. Calcium binding has been shown to contribute to the thermal stability and ligand recognition of CBMs [37]. This predicts that the suitable addition of Ca^2+^ to the reaction system can accelerate the agar hydrolysis while avoiding the inhibition of the enzyme by some harmful ions.

In addition, the crude enzyme of strain ACD-11-B studied in this paper maintains high activity at high salt concentrations (3.0 M), which is related to the fact that the enzyme originates from sea cucumber living in the benthic environment, where the high salinity may coerce symbiotic microorganisms to produce some halophilic or haloduric enzymes. This property makes this enzyme adapt to broad salinity conditions and shows potential commercial value and application prospects.

In the future, strains with high agarase activity, such as strain ACD-11-B, could be subjected to whole genome sequencing to mine their potential agarase gene sequences. The yield of agarase can be increased by heterologous expression, and the purified agarases can be compared with the differences in protein properties and enzymatic properties. For example, Cui et al. heterologously expressed β-agarase derived from *Pseudoalteromonas* sp. Q30F in *Bacillus subtilis*, and further investigated the protein properties as well as the enzymatic properties of agarase by purifying the enzyme [18].

Furthermore, genetic and protein engineering offer promising strategies for agarase modification. Multiple studies have utilized thermal stability prediction tools (e.g., ETSS, PoPMuSiC, HotMuSiC) to design rational mutagenesis schemes, identifying key factors influencing thermostability and critical amino acid residues for mutation. Then, the wild-type enzyme is modified by genetic engineering in order to improve the thermal stability of the enzyme [59]. For example, Zhang et al. applied this approach to engineer the wild-type AgWH50C agarase, generating mutant K621F with a 45% increase in relative activity, an optimal temperature shift from 30 °C to 38 °C, and significantly enhanced thermostability [60]. Additionally, agarase activity modification has emerged as a research frontier. The enzyme’s structure comprises a glycoside hydrolase (GH) module (catalyzing glycosidic bond cleavage) and a non-catalytic carbohydrate-binding module (CBM), both of which are critical for enzymatic activity. The activity of the enzyme can be significantly improved by modifying these two structural domains. Alkotaini et al. successfully expressed the GH16-CBM6-CBM13 structural domain by fusing the CBM6 and CBM13 structural domains of heat-resistant endo-agarase on agarase. The fusion improved the affinity and binding capacity of agarase, thereby greatly increasing the activity of agarose [61].

## 5. Conclusions

In this study, we successfully screened out a strain of *Aspergillus sydowii* ACD-11-B with high-efficiency agar-degrading activity from 18 fungal strains isolated from sea cucumber by the combined use of Gram’s iodine staining and the DNS method. It stably secretes agarase in both solid-state and liquid-state fermentation processes. Zymogram analysis revealed its main extracellular agarases with approximate molecular weights of 180, 95, 43, 33, and 20 kDa, respectively. TLC and HPLC analyses revealed NA2 to be the main agar hydrolytic product by its enzymes. The optimal temperature and pH for the enzymatic hydrolysis of this strain’s crude agarase are 40 °C and 6, respectively. This crude agarase exhibits good stability within the temperature range of 30–40 °C and the pH range of 5.0–7.0. Notably, in the presence of Ca^2+^, Na^+^, and K^+^ ions, the activity of this crude agarase can be enhanced by more than 30%. Conversely, Fe^2+^, Cu^2+^, and CTAB have inhibitory effects on agarase activity. Additionally, it possesses salt-tolerant properties to 0.5–3.0 M NaCl and exhibits strict substrate specificity towards agar.

Collectively, these findings indicate that strain ACD-11-B represents a novel source of agarase. The agarases produced by this strain display favorable enzymatic properties and unique salt-tolerance characteristics, meeting the requirements for potential industrial applications. The intuitive and efficient method for agarase-producing fungi in this study can provide guidance for similar studies.

## Figures and Tables

**Table 1:** The origins, identification, and agarase activities in preliminary screening of the 12 strains.

Strain ID	Isolation Position in Sea Cucumber	Accession Numbers of ITS rDNA Sequences	Closest Fungal Strain	Sequence Identity (%)	Diameter Ratio of Clearance Zone/ Colony
NY5-1	Mucus	OM349543	*Cladosporium colombiae*	99.64%	1.35 ± 0.01
NY-7	Mucus	OM349544	*Cladosporium halotolerans*	100.00%	1.50 ± 0.03
NY-13	Mucus	ON384773	*Aspergillus sydowii*	99.81%	1.57 ± 0.05
CD-1	Intestinal	PV573377	*Penicillium citrinum*	99.14%	1.85 ± 0.03
ACD-2	Intestinal	OM349547	*Penicillium citrinum*	100.00%	1.83 ± 0.07
ACD-3	Intestinal	ON380857	*Cladosporium tenuissimum*	100.00%	1.64 ± 0.06
ACD-5	Intestinal	OM368350	*Penicillium mallochii*	100.00%	1.41 ± 0.04
ACD-7	Intestinal	OM349549	*Cladosporium halotolerans*	100.00%	1.30 ± 0.03
ACD-8	Intestinal	OM349550	*Penicillium citrinum*	100.00%	1.66 ± 0.07
ACD-11-B	Intestinal	PV573475	*Aspergillus sydowii*	99.79%	1.98 ± 0.06
ACD-11-Q	Intestinal	PV573478	*Penicillium citrinum*	100.00%	1.83 ± 0.04
ACD-12	Intestinal	OM349552	*Aspergillus sydowii*	100.00%	1.81 ± 0.02

## Data Availability

The original contributions presented in this study are included in the article. Further inquiries can be directed to the corresponding author.
